# Biointerface topography mediates the interplay between endothelial cells and monocytes†

**DOI:** 10.1039/d0ra00704h

**Published:** 2020-04-06

**Authors:** Yan Liu, Wenshuai Deng, Liangliang Yang, Xiuxiu Fu, Zhibin Wang, Patrick van Rijn, Qihui Zhou, Tao Yu

**Affiliations:** Institute for Translational Medicine, School of Basic Medicine, Qingdao University Qingdao 266021 China qihuizhou@qdu.edu.cn yutao0112@qdu.edu.cn; Stomatology Center, The Affiliated Hospital of Qingdao University Qingdao 266003 China; Department of Echocardiography, The Affiliated Hospital of Qingdao University Qingdao 266003 China; Department of Neurosurgery, The Affiliated Hospital of Qingdao University Qingdao 266003 China; University of Groningen, W. J. Kolff Institute for Biomedical Engineering and Materials Science, Department of Biomedical Engineering, University Medical Center Groningen A. Deusinglaan 1 9713 AV Groningen the Netherlands p.van.rijn@umcg.nl

## Abstract

Endothelial cell (EC) monolayers located in the inner lining of blood vessels serve as a semipermeable barrier between circulating blood and surrounding tissues. The structure and function of the EC monolayer affect the recruitment and adhesion of monocytes, which plays a pivotal role in the development of inflammation and atherosclerosis. Here we investigate the effect of material wrinkled topographies on the responses of human umbilical vein endothelial cells (HUVECs) and adhesion of monocytes to HUVECs. It is found that HUVEC responses are non-linearly mediated by surface topographies with different dimensions. Specifically, more cell elongation and better cell orientation on the wrinkled surface with a 3.5 μm amplitude and 10 μm wavelength (W10) are observed compared to other surfaces. The proliferation rate of HUVECs on the W10 surface is higher than that on other surfaces due to more 5-ethynyl-2′-deoxyuridine (EdU) detected on the W10 surface. Also, greater expression of inflammatory cytokines from HUVECs and adhesion of monocytes to HUVECs on the W10 surface is shown than other surfaces due to greater expression of p-AKT and ICAM, respectively. This study offers a new *in vitro* system to understand the interplay between HUVEC monolayers and monocytes mediated by aligned topographies, which may be useful for vascular repair and disease modeling for drug testing.

## Abstract 2

This study offers a new *in vitro* system to understand the interplay between HUVEC monolayer and monocytes mediated by aligned topographies, which may be useful for vascular repair and disease modeling for drug testing.

## Graphical abstract

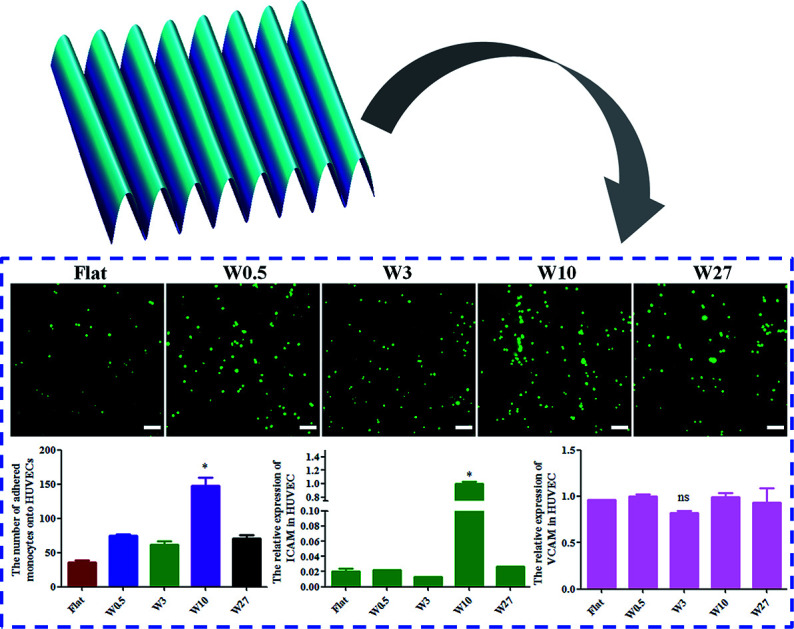

## 1. Introduction

The transport mediated through blood vessels plays a critical role in inflammation, barrier formation, wound healing, as well as tissue morphogenesis and function.^1^ Endothelial cells (ECs) form a continuous inner monolayer lining of blood vessels, and serve as a semipermeable barrier between circulating blood and surrounding tissues as well as the key regulator of inflammation and vascular homeostasis.^2,3^ The adhesion of circulating monocytes to ECs is pivotal for the development of inflammation and atherosclerosis.^4,5^ Later, the attached monocytes migrate into the intima, differentiate into macrophages by macrophage colony-stimulating factor (M-CSF),^6–8^ and regulate vascular remodeling.^9^ Therefore, it is vital to elucidate how to control the interaction between ECs and monocytes.

It has been well-demonstrated that the topography of biomaterial surfaces is regarded as the key regulator for cellular behaviors and function through mechanosensing and mechanotransduction processes, including cell adhesion, morphology, migration, proliferation, differentiation, *etc*^10,11^. Particularly, the ECM structure of vessels alter during EC dysfunction, inflammation, atherogenesis, and the aging process.^12–14^ Importantly, it has been reported that the extracellular matrix (ECM) structure regulated ECs monolayer integrity and permeability.^15^ In addition, vascular ECs are oriented and elongated parallel along the flow direction of blood, whereas disturbed flow results in ECs with polygonal and non-oriented morphology.^10,16,17^ In previous studies, aligned topographic structures (*e.g.*, wrinkles, gratings, fibers) have been used to induce the elongation and alignment of vascular ECs.^16,18,19^ Particularly, wrinkles can efficiently regulate cell adhesion, morphology (*e.g.*, cell shape, spreading, elongation, and orientation), proliferation, the pluripotency or differentiation of stem cells, which have gained much attention as mechano-structural signals in studying cell–material interactions for tissue engineering.^20–23^ However, the effect of biomimetic wrinkled nano/microtopography on the adhesion of monocytes to HUVECs has not been examined closely. Elucidating how directional topography mediates the interplay between ECs and monocytes plays a critical role in improving the development of the vascular graft material.

In this study, we utilized a series of surfaces with various topographical sizes mimicking the structure in the vessel wall to study how matrix topography regulates the interaction between ECs and monocytes. These wrinkled polydimethylsiloxane (PDMS) surfaces with varied dimensions were seeded with HUVECs to study the effect of surface micro/nanotopographies on cellular responses, including cell adhesion, proliferation, morphology changes (*e.g.*, elongation and orientation), the adhesion of monocytes to HUVECs and expression of inflammatory cytokines.

## 2. Materials and methods

### 2.1 PDMS film preparation

The PDMS was synthesized using the elastomer base (prepolymer) and cross-linker (Sylgard 184, Dow Corning) in a ratio of 10 : 1 or 15 : 1 by weight according to their specifications. The mixtures were vigorously stirred with a spatula, degassed under vacuum for 15 min to remove the air bubbles completely, and deposited onto clean 12 × 12 cm polystyrene Petri dish. The PDMS was then cured at 70 °C overnight.

### 2.2 Preparation of wrinkled PDMS substrates

The wrinkled PDMS substrates were fabricated as described previously.^24–26^ Briefly, prepared PDMS substrates were stretched uniaxial in a custom-made stretching apparatus to a strain of 10–30% of their original length. The stretched substrates were oxidized using air plasma (Plasma Activate Flecto 10 USB, maximum intensity) at different pressures and oxidation times. After oxidation, the strain was released which induces the formation of wrinkled topographies (Table 1).

**Table 1:** Conditions for preparing wrinkled PDMS substrates with different dimensions^a^

PDMS substrates	Ratio of prepolymer and cross-linker	Plasma pressure	Oxidation time	Stretched percent (%)
Flat	10 : 1	—	—	0
W0.5A0.05	10 : 1	14 torr	60 s	30
W3A0.7	10 : 1	25 mTorr	20 s	30
W10A3.5	10 : 1	25 mTorr	650 s	20
W27A4.3	15 : 1	25 mTorr	30 min	10

a W and A are the abbreviation of wavelength and amplitude, respectively. And the unit of W and A is μm.

### 2.3 Imprinting

The wrinkled PDMS substrates (Table 1) were used as molds onto which a fresh mixture of elastomer base and cross-linker (in a ratio of 10 : 1 by weight) was poured, followed by curing at 70 °C overnight. Afterwards, the molds were removed and the freshly prepared PDMS substrates baring the imprint were additionally oxidized with air plasma at 500 mTorr for 1 min.

### 2.4 Atomic force microscopy (AFM) characterization

Wrinkled features were measured by AFM (Nanoscope V Dimension 3100 microscope, Veeco, United States) operating with tapping mode in air (model DNP-10 tip). The wavelength and amplitude of wrinkles in obtained AFM images were analyzed using NanoScope Analysis software.

### 2.5 Cell culture

The human umbilical vein endothelial cells (HUVECs) and human acute monocytic leukemia cell (THP-1) were purchased from the Shanghai Institutes for Biological Sciences (Shanghai, China), which were cultured in Dulbecco's Modified Eagle Medium/Nutrient Mixture F-12 (DMEM/F12, Gibco, USA) supplemented with 20% Fetal Bovine Serum (FBS, ExCell Bio, Shanghai, China), 1% penicillin–streptomycin liquid (Solarbio Science & Technology Co., Beijing, China) at 37 °C containing 5% CO_2_. THP-1 was cultured in RPMI 1640 media (Gibco, USA) supplemented with 10% FBS, in a humidified 37 °C and 5% CO_2_ incubator.

### 2.6 Cell adhesion

All circular PDMS substrates (*Ø*14 mm) were sterilized with 70% ethanol and placed in 24-wells. Afterward, HUVECs were seeded onto the substrates above in 24-well plates at a density of 2 × 10^5^ cells per well for cell adhesion. All plates were stored in an incubator at 37 °C and 5% CO_2_ for 24 h. Subsequently, HUVECs were fixed with 4% paraformaldehyde (Solarbio, Beijing, China) at room temperature for 40 minutes. Then, 0.5% Triton X-100 (Solarbio, Beijing, China) was used to increase cell membrane permeability. Finally, the cells were stained by FITC phalloidin (Solarbio, Beijing, China), and the images were captured by fluorescence microscopy (Nikon A1 MP, Japan). ImageJ software was used to measure the cell area, elongation, and orientation. Cell orientation was defined as the angle between the major axis of the cell and the direction of wrinkles.^25^ Cell elongation was quantified as the aspect ratio between the cell length and width measured on the fluorescent F-actin stained cells.

### 2.7 Cell proliferation assay

Cell proliferation was analyzed using the Cell Counting Kit-8 (CCK-8, 7sea, Shanghai, China) according to the manufacturer's protocol. In brief, for 24, 48, and 72 h, CCK-8 solution was added to each well and samples according to dilution ratio of the manufacturer's protocol and the plate was incubated at 37 °C for 30 min. The absorbance was measured by the microplate reader (BioTek, Synergy™ H1/H1M, USA) with the wavelength of 450 nm.

The Cell-Light 5-ethynyl-2′-deoxyuridine (EdU) Apollo567 in Vitro Kit (EdU, RiboBio, Guangzhou, China) was used to measure the specific protein EdU related to cell proliferation rate according to the manufacturer's protocol. Briefly, HUVECs were incubated with EdU solution (50 nmol L^−1^) for 3 h and then stained following the manufacturer's protocol. And all the images of this assay were taken by Fluorescence Microscopy (Nikon A1 MP, Japan).

### 2.8 Monocyte adhesion assay

All circular PDMS substrates (*Ø*14 mm) were sterilized with 70% ethanol and placed in 24-well plates. Afterward, HUVECs were seeded onto the substrates above in 24-well plates at a density of 2 × 10^5^ cells per well for forming HUVEC monolayers. After 24 h, THP-1 cells (3 × 10^5^/well) dyed by carboxyfluorescein diacetate succinimidyl ester (CFSE, MCE, China) were added onto ECs monolayer in 24-well plates, and co-cultured for 4 h. And then, each well was rinsed with PBS for three times and counted the number of THP-1 adhered by HUVEC using the Fluorescence Microscopy (Nikon A1 MP, Japan).

### 2.9 qRT-PCR

Total RNA was extracted from HUVECs by TRI Reagent (Sigma-Aldrich, Cat. No: T9424, USA). The cDNA was synthesized using reverse transcription HiScript® III RT SuperMix for qPCR (Vazyme Biotech, Nanjing, China) and qRT-PCR was used according to the Hieff® qPCR SYBR® Green Master Mix (Yeasen Biotech Co., Ltd., Shanghai, China). The relative expression of IL-1β, THFα, ICAM, VCAM in HUVECs with biomaterial was performed by the 2^−ΔΔ*C*_T_^ method, and were normalized to GAPDH. The sequences of primers in this experiment were as follows: IL-1β, 5′-TCTGCTCGTCTTCCAACATC-3′ (forward) and 5′-AGATCAGCACACTGGAGACG-3′ (reverse);

THFα, 5′-CGACAGCAGCCGCATCTT-3′ (forward) and 5′-CCAATACGACCAAATCCGTTG-3′ (reverse);

ICAM-1 (intercellular cell adhesion molecules), 5′- CAAAGGTGGATCAGATTCAAG-3′ (forward) and 5′- GGTGAGCATTATCACCCAGAA-3′ (reverse);

VCAM-1 (vascular cell adhesion molecules-1), 5′- CAAAGGTGGATCAGATTCAAG-3′ (forward) and 5′- GGTGAGCATTATCACCCAGAA-3′ (reverse);

GAPDH, 5′-CTCGCTTCGGCAGCACA-3′ (forward) and 5′-AACGCTTCACGAATTTGCGT-3′ (reverse).

### 2.10 Western blot analysis

The samples of HUVECs were rinsed with phosphate-buffered saline (PBS) and then add 50 μl lysis buffer (Beijing Solarbio Science & Technology Co., Beijing, China) containing RIPA buffer, 1% PMSF and 0.1% protease inhibitor cocktail in 24-well plate at 4 °C for 20 min, and collected in the 1.5 ml tube. And subsequently, the tube was centrifuged for 15 min at 12000 rpm at 4 °C. The concentration of protein used BCA assay kit (Beijing Solarbio Science & Technology Co., Beijing, China). The sample proteins were separated by 12% SDS-PAGE (EpiZyme, Shanghai, China), and then transferred on the polyvinylidene fluoride (PVDF) membrane (Merck Millopore, USA). The membrane was blocked with 5% defatted milk at room temperature for one hour. After this, the membrane was incubated by Rabbit Anti-phospho-AKT1 (Thr34) polyclonal antibody (Bioss, Beijing, China) with a dilution of 1 : 1000 and incubated by GAPDH (Cell Signaling Technology, USA) with a dilution of 1 : 1000. In the next step, the membrane was rinsed three times using Tris–HCl buffered saline with Tween 20 (TBST), and it was incubated by each corresponding secondary antibody and washed three times. Subsequently, the membrane was visualized using a Fusion FX7 Multifunction imaging system (Vilber, France). And the p-AKT and the GAPDH band were analyzed using Image J (Java 1.8.0-172).

### 2.11 Statistical analyses

The data were presented as mean values ± standard deviation, and all the data performed three independent experiments. Statistical analysis was performed using GraphPad Prism 5 software. All data were analyzed using one-way analysis of variance (ANOVA) with Tukey's test to determine differences between groups. A value of *p* < 0.05 was considered to be statistically significant.

## 3. Results and discussion

### 3.1 PDMS wrinkle formation and characterization

The wrinkled PDMS surfaces with various dimensions were prepared by adjusting key parameters in our previously reported strain-oxidation-release procedure,^24,26^ including the ratio of cross-linker to pre-polymer, the percentage of unidirectional strain, the operating pressure, and the plasma treating time (Table 1). It was found that wrinkle amplitude and wavelength increased with increasing the ratio of pre-polymer to cross-linker, operating pressure, and plasma treating time, but decreasing the percentage of unidirectional strain. The wrinkles prepared had wavelengths in the range between 0.5 and 25.0 μm, amplitudes in the range of 0.05 to 4.30 μm and aspect ratios in the range of 0 to 0.34. However, the parameters above can also affect the physicochemical characteristics of PDMS, which could interfere with cell experimental results. As a consequence, an improved fabrication strategy was required to exclude all effects other than topography.

Using the wrinkled PDMS surfaces above as templates, the imprinted wrinkle structure surfaces were made by pouring the mixture of prepolymer and cross-linker at a 10 : 1 ratio by mass as well as curing at 70 °C for overnight (Fig. S1†), which can exclude any chemical or stiffness variations (The reference submitted to Biomaterials Science). The dimensions of wrinkled PDMS surfaces were measured by AFM. It was found that the PDMS without any stretching led to a smooth surface. After imprinting on the wrinkled PDMS samples as illustrated in Table 1, wrinkled topographies with various dimensions (*i.e.*, W0.5A0.05, W3A0.7, W10A3.5 and W27A4.3) were obtained (The reference submitted to Biomaterials Science), which were designated as W0.5, W3, W10, and W27, respectively. The size range of designed wrinkles from nanometer to a few microns to dozens of microns would contribute to comprehensively explore how wrinkled topography regulates the interplays between endothelial cells and monocytes.

### 3.2 HUVEC adhesion is affected by wrinkled topographies

**Fig. 1:**
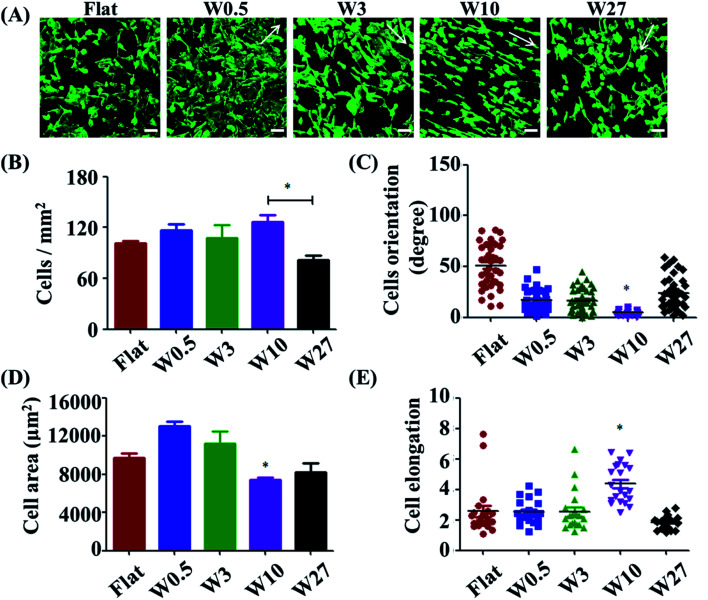
(A) Fluorescence microscopy images of HUVEC adhesion on wrinkled PDMS substrates with various dimensions after being cultured for 1 d. The green staining is F-actin. Scale bar: 20 μm. (B–D) Dependence of cell density (B), spreading area (C) and elongation (D), and orientation (E) on wrinkled PDMS substrates with various dimensions after being cultured for 24 d, respectively. Data are reported as mean ± standard deviation (SD) (*n* = 100–150 cells).

Cell adhesion is considered as the initial response of the cell with its surrounding biomaterial, which precedes all other cellular events, such as spreading, migration, proliferation, and differentiation.^27,28^ To investigate the early cell behavior on the wrinkled PDMS surfaces, HUVECs were seeded on these substrates and allowed to attach and spread for 24 h. HUVEC adhesion on the respective surfaces was investigated with a double-label fluorescence staining of actin (cytoskeleton) and cell nucleus. As shown in Fig. 1A, there were large differences observed between the wrinkled surfaces and flat control. Particularly, HUVECs were better oriented and more elongated on the W10 surface as compared to those on other surfaces.

For a better understanding of initial cell response on various substrates, the number of attached cells, cell area, and elongation were determined by a quantitative analysis of the positively stained cells (Fig. 1B–D). It was found that more cells attached on the flat, W0.5, W3, W10 surfaces than that on the W27 surface (Fig. 1B). Cell area and elongation initial increased and then decreased with increasing wrinkle dimensions from flat to W27 (Fig. 1C and D). Cell orientation was determined to measure the angle of a cell relative to the direction of the wrinkles using ImageJ software (Fig. 1E). Fig. 1E shows that the orientation of cells also first increased and then decreased with increasing wrinkle dimensions from flat to W27. These results indicate a positive correlation of cell orientation with cell elongation, which is consistent with the literatures.^18,19,25^ It was reported that cell orientation and elongation play a critical role in cell responses, *e.g.*, cytoskeleton reorganization, membrane protein relocation, migration direction, ECM remodeling, functionalization, differentiation, and even tissue regeneration.^29–31^ Therefore, it is vital to build cell alignment *in vitro* using structured substrates.

### 3.3 HUVEC proliferation is affected by wrinkled topographies

**Fig. 2:**
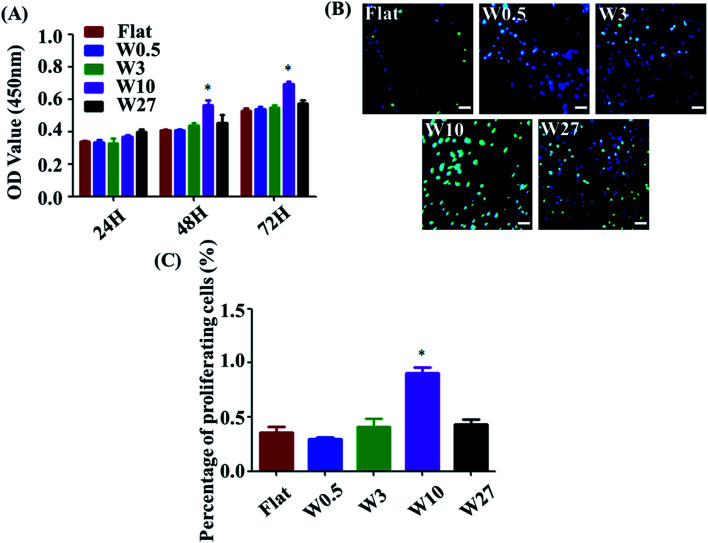
(A) Cell proliferation of HUVECs cultured on different wrinkled PDMS substrates. Data are reported as mean ± standard deviation (SD). (*N* = 3) (*p < 0.05). (B) Fluorescent images of HUVECs cultured on different wrinkled PDMS substrates for 24 h. Scale bar: 20 μm. Green: EdU; blue: nucleus. (C) EdU per cell on different wrinkled PDMS substrates for 24 h (*N* = 3). Data reported as mean ± standard deviation (SD). (*p < 0.05).

Measuring cell proliferation over the culture time is a critical step of biocompatibility evaluation to test if a material property is appropriated for biomedical applications or not. To investigate cell proliferation, HUVECs were cultured onto the wrinkled PDMS substrates with different topography dimensions for 24, 48, and 72 h and characterized using a CCK-8 assay. As indicated in Fig. 2A, all substrates display well-supported cell proliferation for up to 72 h. Particularly, cell number on the W10 surface for 48 and 72 h cultures was more than that on the other surfaces. Additionally, a key protein marker (EdU) for cell proliferation was detected. The expression of EdU on the W10 surface for 24 h cultures was much higher than that on other surfaces from the fluorescent images and quantitative data (Fig. 2B and C). The data suggest that the difference in cell number on topographical substrates is mainly attributed to the difference in their proliferation rate and not the initial attached cell number. Importantly, the W10 surface supported HUVECs to populate better, which is beneficial for further ECM secretion as well as tissue repair and regeneration.

### 3.4 The inflammatory response of HUVECs is affected by wrinkled topographies

**Fig. 3:**
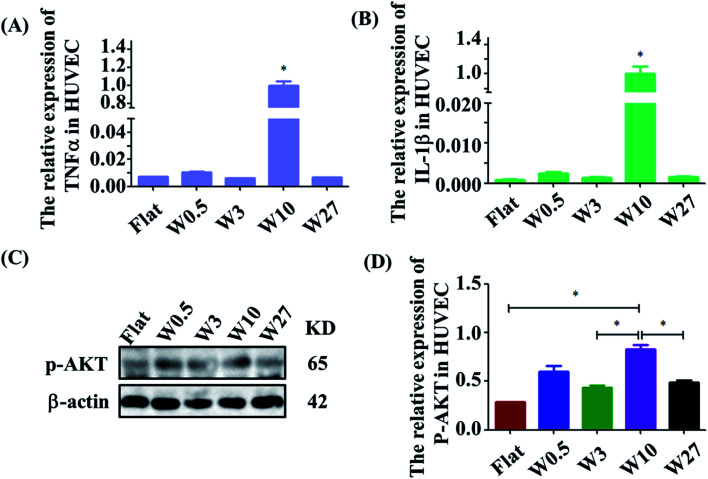
(A and B) Real-time PCR analysis of relative mRNA expression levels of IL-1β and TNF-α genes on wrinkled topographies over 24 h of culture. (C) Western blot analysis of p-AKT protein expression on wrinkled topographies over 24 h of culture. (D) Real-time PCR analysis of relative mRNA expression levels of p-AKT on wrinkled topographies over 24 h of culture.

The effect of wrinkled topographies on the endothelial secretion of inflammatory cytokines was tested. It was found that aligned topographical sizes indeed affected the endothelial secretion of inflammatory cytokines. The gene expression level of IL-1β and TNF-α on the W10 surface was significantly higher than that on other surfaces (Fig. 3A and B). Then, we further studied the underlying mechanism of the interaction between material topography and endothelial cells. As reported, AKT/NF-κB signaling was important to inflammation and adhesion. It was found that HUVECs on the W10 surface had higher gene and protein expression of p-AKT than that on other surfaces from the images and quantitative data (Fig. 3C and D). These results indicate that directional topography significantly mediated the inflammation response of HUVECs, which provides an insight for health blood vessel regeneration.

### 3.5 The adhesion of monocytes to HUVECs is affected by wrinkled topographies

**Fig. 4:**
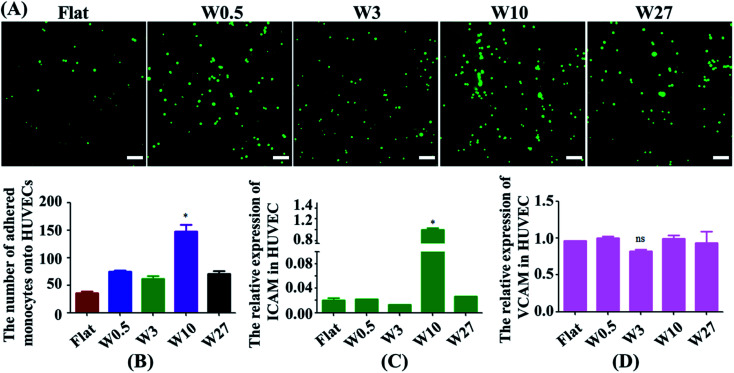
(A) Fluorescent images of the adhesion of monocytes to HUVECs mediated by wrinkled topographies. Scale bar: 20 μm. (B) Statistical analysis of monocyte adhesion on HUVECs (*N* = 3). *p < 0.05. (C and D) Real-time PCR analysis of relative mRNA expression levels of ICAM-1 and VCAM-1 genes on wrinkled topographies over 24 h of culture.

Monocytes in blood circulation were collected by activated ECs followed by monocyte adhesion to the EC monolayer, which is highly related to the generation of atherogenesis.^32,33^ Hence, a monocyte adhesion assay on HUVEC monolayers was carried out (Fig. 4A). ECs were cultured on the substrates until they generated a monolayer. Fluorescently labeled monocytes were then seeded onto EC monolayer, and the adhesion of the monocytes was quantified after 24 hours (Fig. 4B). HUVEC monolayers cultured on wrinkled surfaces displayed an increase in monocyte adhesion compared with the flat control. Interestingly, HUVECs on the W10 surface exhibited higher monocyte adhesion as compared to other surfaces from the fluorescent images and quantitative data (Fig. 4A and B).

Then, we explored why material topography mediated the adhesion of monocytes to ECs. As it was demonstrated that both ICAM-1 and VCAM-1 were closely related to the interplay of ECs and monocytes,^34,35^ the endothelial expression of ICAM-1 and VCAM-1 was tested in ECs cultured on PDMS substrates with different dimensions. The gene expression of ICAM-1 and VCAM-1 was performed by RT-qPCR. The endothelial ICAM-1 (Fig. 4C) showed higher mRNA expression on the W10 surface as compared to those on other surfaces. However, the endothelial VCAM-1 had no significant difference on all surfaces (Fig. 4D). Therefore, more monocyte adhesion is closely related to the increased ICAM-1 expression.

## 4. Conclusions

In summary, wrinkled PDMS substrates with various dimensions from nano to micro level were prepared using the imprinting strategy. It was found that HUVEC responses were non-linearly mediated by surface topographies. HUVECs grown on the W10 surface were stimulated to have the highest cell orientation, the highest cell elongation and proliferation rate than other surfaces. Importantly, more expression of inflammatory cytokines and adhesion of monocytes to HUVECs on the W10 surface is found compared to other surfaces mediated by p-AKT and ICAM, respectively. This work provides a new *in vitro* system to understand how HUVEC monolayer mediated by aligned topographies interacts with monocyte adhesion.

## Conflicts of interest

The authors declare no conflict of interests.

## Supplementary Material

RA-010-D0RA00704H-s001
