# Supplementary material for: Biointerface topography mediates the interplay between endothelial cells and monocytes
Source: RSC Adv. 2020 Apr 6;10(23):13848–54. doi: 10.1039/d0ra00704h (PMC9051607; doi:10.1039/d0ra00704h)
Supplement: RA-010-D0RA00704H-s001 [file RA-010-D0RA00704H-s001.pdf]

Electronic Supplementary Material (ESI) for RSC Advances.
This journal is © The Royal Society of Chemistry 2020

Biointerface topography mediates the interplays between endothelial cells and monocytes

Yan Liu [a], Wenshuai Deng [d], Liangliang Yang [e], Xiuxiu Fu [c], Zhibin Wang [c], Patrick van Rijn *[e], Qihui Zhou *[a,b], Tao Yu *[a,c]

Author Affiliations:

[a] Institute for Translational Medicine, School of Basic Medicine, Qingdao University, Qingdao, 266021, China.

[b] Stomatology Center, The Affiliated Hospital of Qingdao University, Qingdao, 266003, China.

[c] Department of Echocardiography, The Affiliated Hospital of Qingdao University, Qingdao, 26603, China.

[d] Department of Neurosurgery, The Affiliated Hospital of Qingdao University, Qingdao 266003, China.

[e] University of Groningen, W.J. Kolff Institute for Biomedical Engineering and Materials Science, Department of Biomedical Engineering, University Medical Center Groningen, A. Deusinglaan 1, 9713 AV, Groningen, the Netherlands.

*Corresponding author:

Patrick van Rijn, Ph.D., Associate professor of Biomedical Engineering, Email: p.van.rijn@umcg.nl.

Qihui Zhou, Ph.D., Associate professor of Biomaterials, Phone: +86-17660670299, Email: qihuizhou@qdu.edu.cn.

Tao Yu, Ph.D., Associate professor of Basic Medicine, Email: yutao0112@qdu.edu.cn.

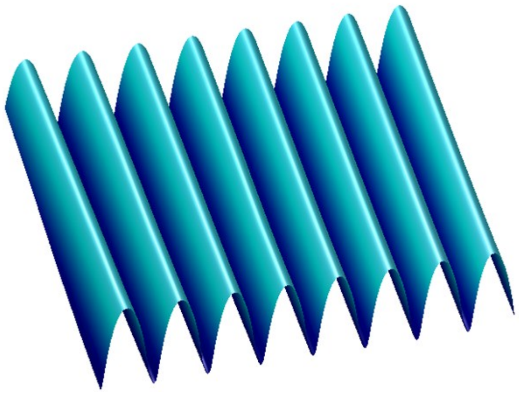

Figure S1. Imprinted PDMS wrinkles using the wrinkled PDMS surface as the template.
